# Supplementary material for: Cryptic population structure and insecticide resistance in Anopheles gambiae from the southern Democratic Republic of Congo
Source: Sci Rep. 2024 Sep 18;14:21782. doi: 10.1038/s41598-024-70885-7 (PMC11410927; doi:10.1038/s41598-024-70885-7)
Supplement: Supplementary file 1 — Supplementary Figures. [file 41598_2024_70885_MOESM1_ESM.docx]

**SUPPLEMENTARY FIGURES**


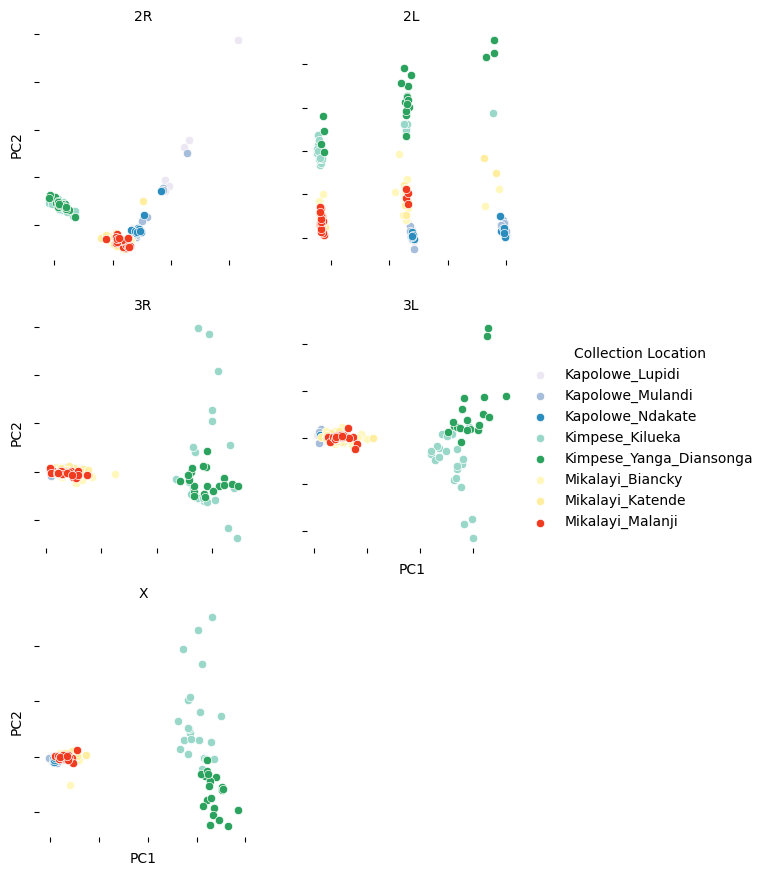


**Figure S1** – Principal component analysis (PCA) of all 165 sequenced *An. gambiae* samples. Plots are panelled by chromosome arm. Points, denoting individuals, are coloured by collection location, indicated in the key (which indicates collection district and collection site).

**
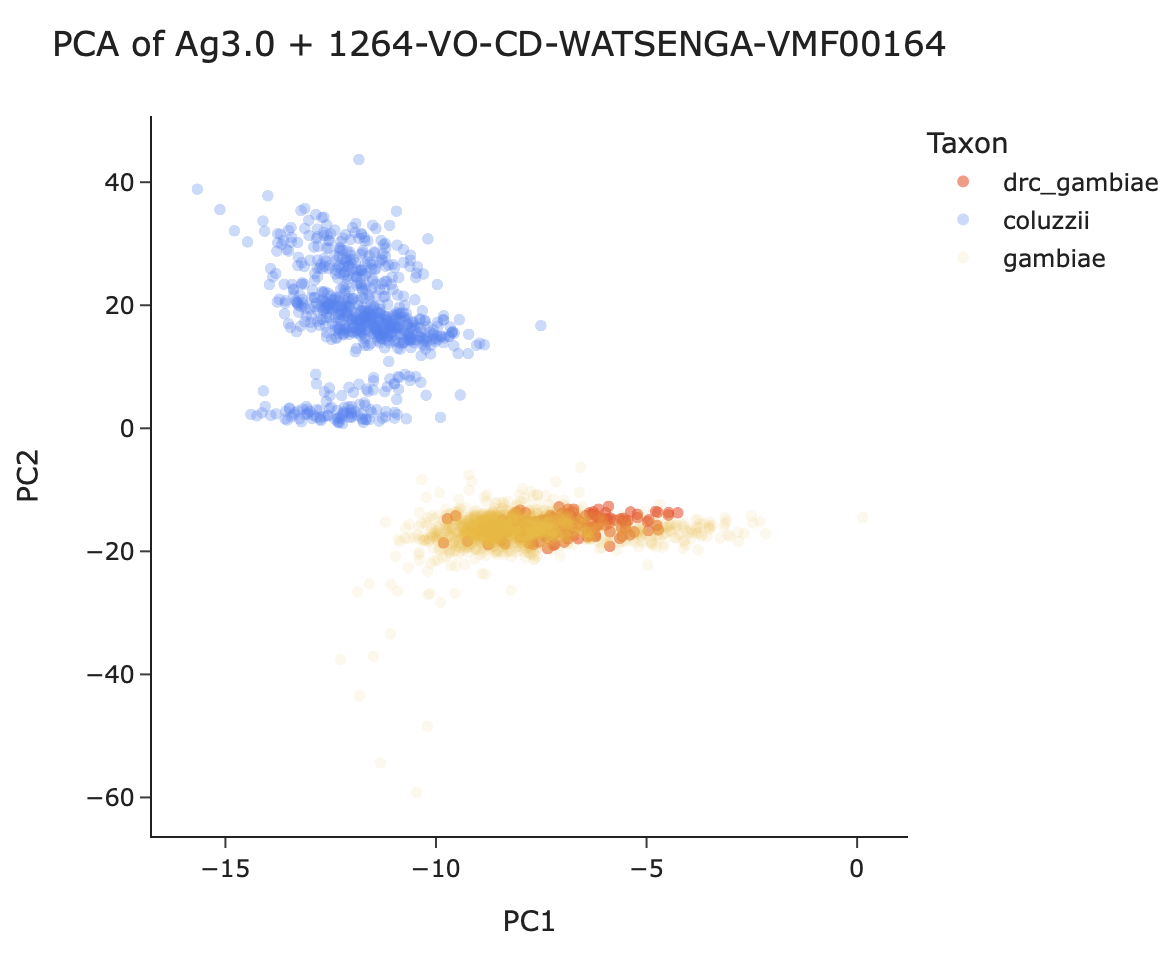
**

**Figure S2 –** Principal component analysis (PCA) of all 165 sequenced *An. gambiae* samples, compared to *An. gambiae s.s.* and *An. coluzzi* samples from the *An. gambiae* 1000 genome project phase 3.0 release. Point colour indicates taxon.
